# Myanmar immigrant women’s perceptions, beliefs, and information-seeking behaviors with nutrition and food practices during pregnancy in Thailand: a qualitative study

**DOI:** 10.1186/s12939-024-02240-1

**Published:** 2024-08-07

**Authors:** Sasitara Nuampa, Pornnapa Tangsuksan, Kwanchit Sasiwongsaroj, Rudee Pungbangkadee, Somsiri Rungamornrat, Nuntiya Doungphummes, Sittiporn Netniyom, Crystal L. Patil

**Affiliations:** 1 Department of Obstetric and Gynaecological Nursing, Faculty of Nursing, Mahidol University, Bangkok, Thailand; 2 Department of Cultural Studies, Research Institute for Languages and Cultures of Asia, Mahidol University, Nakhon Pathom, Thailand; 3 Department of Pediatric Nursing, Faculty of Nursing, Bangkok, Mahidol University, Bangkok, Thailand; 4 Department of language and intercultural Communication, Research Institute for Languages and Cultures of Asia, Mahidol University, Nakhon Pathom, Thailand; 5 Center for Bharat Studies, Research Institute for Languages and Cultures of Asia, Mahidol University, Nakhon Pathom, Thailand; 6 Department of Health Behavior and Biological Sciences, School of Nursing, University of Michigan, Ann Arbor, Michigan USA

**Keywords:** Immigrant, Myanmar, Nutrition, Pregnancy, Qualitative study

## Abstract

**Background:**

Although nutrition is an essential contributor to the quality of pregnancy outcomes, little is known about the experiences and influences affecting dietary behaviors during pregnancy among migrant women, particularly those from Myanmar, the largest immigrant population in Thailand. To fill this gap, we conducted a descriptive qualitative study to explore Myanmar immigrant women’s perceptions, beliefs, and information-seeking behaviors concerning nutrition and food practices during pregnancy.

**Methods:**

We conducted focus group discussions (FGDs) with fifty Myanmar immigrant pregnant women aged 18–45 years across all trimesters, who were recruited using purposive sampling from a public tertiary hospital. The FGDs were conducted in Thai or Myanmar using semi-structured guides that probed women’s pregnancy perceptions and experiences about nutrition and food patterns during pregnancy. The FGDs were audio-recorded, translated, and transcribed. Direct content analysis was used to guide the analysis through an ecological perspective framework.

**Results:**

The seven FGDs with fifty women revealed four major themes involving perceptions, beliefs, and information-seeking behaviors. The qualitative results consisted of (1) a positive attitude toward better changes under difficult conditions (setting goals for infant health; uncertainty about changes); (2) beliefs about eating patterns and dietary practices during pregnancy (taboos aimed at protecting women’s health and ensuring safe childbirth; taboos aimed at guaranteeing infant safety); (3) limited access to appropriate information about nutrition (unclear dietary information from healthcare providers; ease of learning from experiences in informal social networks); and (4) difficult living conditions in a non-native setting (work-related influences on dietary behaviors; lack of comprehensible language to gain food literacy). In addition, the results were highlighted across four levels of ecological perspectives.

**Conclusions:**

Immigrant pregnant women are a vulnerable population that should be treated with equity to ensure quality of life through optimal nutrition throughout pregnancy. Respectful care requires that healthcare providers develop culturally sensitive nutrition interventions to increase nutrition literacy, accessibility, and pregnancy outcomes.

## Background

Proper nutrition during pregnancy is a major public health concern [1]. Malnutrition affects more than half of women in many low- and middle-income countries (LMICs), with the poorest being most vulnerable [1, 2]. Improving nutrition sits at the core of global development and is central to achieving the Sustainable Development Goals [3]. The UNICEF Nutrition Strategy 2020–2030 guidance mentions and supports securing nutritious and safe diets, essential nutrition services, and positive nutrition practices for women in all contexts [4]. However, pregnant populations have been found to have poor intake of the recommended food groups [5, 6]. Most pregnancy-related dietary modifications involve avoiding alcohol, caffeine, and foods with safety concerns rather than improving overall diet quality [5].

The growth of migration has been robust worldwide, reaching 281 million international migrants in 2020, or accounting for 3.6% of the global population [7]. Previous studies have reported that migrant women are more likely to have suboptimal diets and experience a higher prevalence of diet-related noncommunicable diseases [8, 9]. Migrant women had higher odds of inadequate gestational weight gain nearly two times (OR, 1.97; 95% CI, 1.87–2.07) and higher odds of obesity (OR, 1.40; 95% CI, 1.35–1.44) compared with native-born women [9]. Regarding the pregnancy period, the literature shows that migrant women in high-income countries have more adverse pregnancy outcomes than native women [10, 11]. A study in Sweden showed that migrant women were nearly three times as likely to be underweight and twice as likely to experience inadequate gestational weight gain compared to non-migrant women [9]. Inadequate gestational weight gain is associated with an increased risk of preterm delivery [12, 13], small-for-gestational-age fetuses and low-birthweight infants [12]. A study in an urban area of South Africa that explored migrant perspectives on food during pregnancy reported that migrant women tended to consume calorie-dense, nutrient poor fast foods, and junk foods, and framed diet during pregnancy in terms of cravings and feeling satiated, not in terms of healthy foods [14]. Excessive gestational weight gain is associated with increased risks of cesarean delivery, pregnancy-related hypertension [15], diabetes [16], large-for-gestational-age infants, as well as overweight and obese children [17].

Although there are studies examining maternal diet among migrants in high-income settings [18], understanding of the connections between migration and maternal nutrition in low- and middle-income countries (LMICs) remains limited. This gap persists despite migrants constituting a significant and frequently vulnerable population worldwide. The global migrant population is 73 million people residing in LMICs [19]. Thailand is one of the most significant recipient countries for international migrants and refugees in Southeast Asia, with 3.6 million workers from the bordering countries of Myanmar, Lao PDR, and Cambodia [20]; 1.7 million, or 73%, of all documented migrants in Thailand are from Myanmar [21]. Many migrant workers are employed in the informal sector in low-skilled what are called “3D jobs” (dirty, dangerous, and demeaning/demanding), such as agriculture, fisheries, domestic work, manufacturing, and construction [22]. The challenges in health disparities are related to limited access to the healthcare financial coverage system, financial risk protection, health care services, and access to safe, effective, quality, and affordable essential medicine [23].

Half of all migrant workers in Thailand are women [22]. Women migrants face additional challenges in accessing sexual and reproductive health services and are at a higher risk of maternal mortality [24]. Previous studies have shown several challenges during pregnancy among the migrant population on the Myanmar-Thailand border, including difficulty accessing care [25], language limitations, inability to pay for the cost of care [26], and low literacy [27]. In addition, migrants encounter significant challenges to their social well-being, particularly among pregnant women, who face limited options for adequate prenatal care and struggle with restricted access to nutritious foods [28, 29]. Both the quantitative and qualitative findings from a mixed-methods study with 388 pregnant migrants living on the Myanmar-Thailand border showed that pregnant migrant women had limited awareness about nutrition and healthy dietary practices [30]. According to a study in Thailand summarizing nutrition trends among pregnant women along the Thailand-Myanmar border over 30 years, the proportion of women with low BMI in the first trimester decreased from 23.1 to 20.2%, whereas high BMI increased markedly from 12.3 to 28.4% in migrants. In addition, low BMI is commonly found among Burmese migrants [31]. A cross-sectional study among Myanmar immigrants living in Thailand reported that 41% of the 186 children who were born premature were also underweight at 2 years of age, which shows the intergenerational and long-term effects resulting from poor nutrition [32]. Pregnant migrants, particularly those from Myanmar, face several inequities during the antenatal period and difficulties with food behavior during this vital period.

Although it is well established that poor nutrition negatively affects pregnancy outcomes, Myanmar migrant women still encounter difficulties with antenatal care services. The challenges include language and cultural barriers, legal and administrative issues, inadequate health facilities and staff, stigma and discrimination [33, 34]. There is little known about the perceptions and practices of Myanmar migrant women living in urban areas of Thailand concerning nutrition and food during pregnancy. To fill this gap, the objective of this study is to explore Myanmar immigrant women’s perceptions, beliefs, and information-seeking behaviors with nutrition and food practices during pregnancy in Thailand through an ecological perspective for health promotion [35].

## Methods

### Study setting

This study took place in Samut Sakhon Province, which is located southwest of Bangkok, Thailand. Samut Sakhon Province has numerous sea product-processing facilities and is one of the most attractive destinations for migrant jobseekers [36]. A 2023 situation report indicated that 91% of the 241,954 migrant workers in the area were from Myanmar. Moreover, birth rates continued to increase to 4,775 in 2022. According to a demographic data survey among adults in assessed households, 29% had less than a primary school education, and 51% had completed primary school. In employment, the most common work sectors included food production (42%), manufacturing (36%), and agriculture (11%). Regarding access to health care, 17% of the households lacked any insurance [37]. Myanmar pregnant women in Samut Sakhon Province, which has the highest migrant population, may also live with difficult health conditions.

### Study design and participants

This qualitative study was part of the major project study, “A Strategy Development for Moving Forward the Nutrition Literacy During Pregnancy 270 Days of Miracle Life: Phase 1 Situation Analysis.” Focus group discussions (FGDs) were used to explore Myanmar immigrant women’s perceptions, beliefs, and information-seeking behaviors about nutrition and food practices during pregnancy. The participants for this study were recruited from an antenatal clinic at a public or government tertiary hospital. With the assistance of nurses and Myanmar translators, pregnant women were approached by research assistants to learn about the study. Those who agreed to participate provided written, informed consent. The sampling approach aimed to select participants with diverse cultures and beliefs. We used simple random selection to select one tertiary hospital in the Samut Sakhon Province. Moreover, we purposively sampled 50 pregnant women who were Myanmar immigrants aged 18–45 years and had singleton pregnancies. The participants might possess fluency in Thai; however, we prepared Myanmar translators during data collection. This paper was reported according to the Standards for Reporting Qualitative Research (SRQR) Guidance [38].

### Researcher characteristics and reflexivity

The focus group discussions were conducted by the researchers with more than 5 years of experience in qualitative research who were not affiliated with the agencies where data collection took place. However, the researchers had experience working in antenatal care units and had provided services to pregnant migrant women in tertiary hospitals for over 10 years. One of the researchers was of Mon descent, an ethnic group of Myanmar, and fluent in the Mon language. For the discussions with pregnant women, the women could speak either Thai or Burmese, as there was a female interpreter assigned to the group to help the participants feel more comfortable. In addition, the research assistants responsible for field note-taking were also women. The activities were arranged after medical check-ups to ensure that the participants did not feel pressured.

### Data collection

Three authors (SN, PT, and RP) conducted seven audio-recorded FGDs (60–90 min) between October and December 2022 with the assistance of two Myanmar translators and two research assistants. Each FGD took place in a private setting that was convenient for the women. In the first 10–15 min, each participant completed a 14-item Personal Information Form to capture demographics, reproductive history, and delivery characteristics. The FGD interview guide, informed by the health literacy literature and a literature review about nutrition and food beliefs, perceptions, and practices during pregnancy [39–42], had questions designed to explore (1) perceptions about nutrition during pregnancy; (2) information-seeking behaviors; and (3) factors affecting eating behaviors.

### Analysis

The audio recordings of each FGD were transcribed into Thai, and direct content analysis was used [43, 44]. Two authors (SN and PT) read several transcripts and took notes independently to develop an agreed upon preliminary codebook. Data collection and analysis were conducted concurrently to ensure that new concepts emerging from the FGDs could be explored in detail. In addition, field notes were taken immediately after the interviews to inform the researchers of important issues based on the interviewer’s reactions. Next, codes were developed deductively and inductively, and the transcripts were coded line-by-line, emphasizing specific words from the text that appeared to identify the key themes. Finally, the researchers looked over the coded data extracts for each topic determine whether the issues appeared to form a coherent pattern [45]. During the analysis process, the researchers met regularly to discuss the interpretive results by team consensus (SN, PT, KS, RP, and SR). In addition, the results of this study were analyzed from an ecological perspective on health promotion in which health-promoting behavior focused attention on both individual and social environment perspectives [35].

To ensure trustworthiness, an audit trail was maintained using audio recordings, allowing the research team to repeatedly check the data throughout the study. Field notes were taken immediately after the interviews to accurately capture contextual information. Rigorous content analysis and peer debriefing with the research team were conducted to establish credibility. Member checking was incorporated during the interviews to confirm the participants’ intended meanings, further ensuring the credibility of the results [43].

## Results

### Sociodemographic characteristics and pregnancy data of the FGD participants

The participants’ sociodemographic and reproductive characteristics are in Table 1. On average, the participants were 28.84 (SD 5.84) years old. Educational levels were low, with most (68%) having completed primary school. The average family income was 16,820 baht per month (SD 5,391.73) (approximately 480 USD; exchange rate = 35 baht per USD), within a range of 9,000 to 28,000 baht (257–800 USD per month). Regarding pregnancy information, the average number of children was 1.82 (SD 0.91), while the average gestational age was 21.94 weeks (SD 11.18). None of the women attended parenting classes during antenatal care.

**Table 1:** Sociodemographic characteristics and pregnancy data of the FGD participants

Characteristics	*n* (%)
Age (years)	
< 35 ≥ 35	40 (80.0) 10 (20.0)
Religion	
Buddhism Islam	48 (96.0) 2 (4.0)
Marital Status	
Married Divorce	49 (98.0) 1 (2.0)
Educational Status	
None Primary School Middle School High School	5 (10.0) 34 (68.0) 5 (10.0) 6 (12.0)
Family Income (baht per month)	
< 15,000 ≥ 15,000	20 (40.0) 30 (60.0)
Family Income Status	
Sufficient Insufficient	36 (72.0) 14 (28.0)
Family Characteristics	
Nuclear Family Extended Family	40 (80.0) 10 (20.0)
Gravida	
Primiparous Multiparous	23 (46.0) 27 (54.0)
Gestational Age (weeks)	
< 14 14–28 > 28	21 (42.0) 13 (26.0) 16 (32.0)
First ANC Visit	
≤ 12 Weeks’ Gestational Age > 12 Weeks’ Gestational Age	37 (74.0) 13 (26.0)
BMI (kg/m^2^) before Pregnancy	
< 18.5 18.5–22.9 ≥ 23	3 (6.0) 25 (50.0) 22 (44.0)

### Experiences of Myanmar immigrant women with nutrition and food practices during pregnancy

The pregnant women in this study disclosed their experiences with dietary practices, which had important changes compared to the pre-pregnancy period. However, the immigrant life context meaningfully affected their adaptations and decisions about food practices. Four themes emerged about the perceptions, beliefs, and information-seeking behaviors concerning nutrition and food practices among Myanmar immigrant pregnancy, as shown in Table 2.

**Table 2:** Illustrative quotes by theme

Themes and Sub-themes	Sample Quotes
**Perceptions of Dietary Changes During Pregnancy**	
**Theme 1: Positive Attitude Toward Better Changes under Difficult Conditions**	
Sub-theme 1.1: Setting Goals for Infant Health	*“My baby stays inside me; I have to try to eat good foods. I drink more milk…”* (Woman in First Trimester, Group 2)
Sub-theme 1.2: Uncertainty about Changes	*“… Some products have the Burmese language on the labels*,* but only a little bit… However*,* most products have the Thai language on the labels*,* which I cannot read.”* (Woman in Second Trimester, Group 3)
**Beliefs about Healthy Eating During Pregnancy**	
**Theme 2: Beliefs on Eating Patterns and Food Practices During Pregnancy**	
Sub-theme 2.1: Taboos aimed at Protecting Women’s Health and Ensuring Safe Childbirth	*“My grandmother says it is taboo to have lunch with her*,* because she is afraid*,* it’ll cause an obstruction of labor. So*,* I need to drink some milk and eat some bread instead.”* (Woman in First Trimester, Group 1)
Sub-theme 2.2: Taboos aimed at Guaranteeing Infant Safety	*“In the beginning*,* I really wanted to eat spicy foods*,* but my mom stopped me because spicy foods will make my baby feel hot and burned*,* or might even cause abortion.”* (Woman in Second Trimester, Group 4)
**Information-seeking Behavior Regarding Pregnancy Diet**	
**Theme 3: Limited Access to Appropriate Information about Nutrition**	
Sub-theme 3.1: Unclear Dietary Information from Healthcare Providers	*“Sometimes I cannot understand what nurses are saying to me; I need some leaflets to read again at home… The difficulty of understanding makes me more stressed.”* (Woman in First Trimester, Group 2)
Sub-theme 3.2: Ease of Learning from Experiences in Informal Social Networks	*“I must believe her [grandmother]; I cannot read Thai…she has a lot of experience with this [dietary behavior during pregnancy].”* (Woman in First Trimester, Group, 1)
**Theme 4: Difficult Living Conditions in a Non-Native Setting**	
Sub-theme 4.1: Work-related Influences on Dietary Behaviors	*“I have to eat good foods for being strong to work”* (Woman in First Trimester, Group 2).
Sub-theme 4.2: Lack of Comprehensible Language to Gain Food Literacy	*“…a lot of data on the internet and social media. I can’t decide and have realized that I spend a lot of time at work. I think I’ll ask my friends who have previous experience to be certain and save time.”* (Woman in Second Trimester, Group 5)

### Perceptions of dietary changes during pregnancy

#### Theme 1: positive attitude toward better changes under difficult conditions

All the women perceived a positive attitude toward changing their eating behavior to be healthier during pregnancy. They admitted that they tried to adjust healthy foods for themselves and particularly valued changing food practices for infant health. They made changes, even though they still had some questionable food choices that might make them skip healthy foods sometimes. The following two sub-themes were presented:

#### Sub-theme 1: setting goals for infant health

Nearly all the women expressed that they had improved their dietary behaviors positively through increasing healthy foods (e.g., milk, meat, fish, fruits, eggs) and weaning themselves off harmful foods (e.g., raw meat, alcohol, caffeine, soft drinks). The main reason for encouraging them to eat healthy foods and patterns was their babies’ health. They perceived that maternal foods could transfer directly to their babies. For example, a pregnant woman in the first trimester had morning sickness symptoms. She said that she had to increase some milk and fruits, even though she did not like these kinds of foods.

*“My baby stays inside me; I have to try to eat good foods. I drink more milk*,* 2 glasses per day*,* and increase some fruits such as watermelon or oranges.” (Woman in First Trimester*,* Group 2)*.

One pregnant woman described her changing food pattern from two meals to three meals because of her baby’s health.

*“I try to increase my morning meal and set the exact time for my diet every day*,* which differs from pre-pregnancy. Moreover*,* I drink more milk and eat fruits*,* as well as fish and meat for my baby. (Woman in Second Trimester*,* Group 3)*

#### Sub-theme 2: uncertainty about changes

Some pregnant women perceived the difficulty of using Thai for communication and reading. This limitation could make them doubtful and unaware of the food products and their detailed descriptions. In particular, the source of food products was likely located in the local markets, and their education levels might have prohibited them from clearly understanding the information provided. For example, one woman described her difficulty making decisions about food products in Thai.

*“Normally*,* I go to the local market to buy food. Some products have the Burmese language on the labels*,* but only a little bit… However*,* most products have the Thai language on the labels*,* which I cannot read.”* (Woman in Second Trimester, Group 3).

Moreover, one woman admitted to living alone in a nuclear family. When she could not read and did not understand, she was unable to ask anyone. Thus, she often guessed some food products that she would like to buy and test.

*“I do not have any family members here. When I am not sure about any foods*,* I just try to eat a little and wait for the results.” (Woman in Third Trimester*,* Group 6)*.

### Beliefs about healthy eating during pregnancy

#### Theme 2: beliefs about eating patterns and Food practices during pregnancy

The pregnant women in this study disclosed similar beliefs about diet during pregnancy. The beliefs were transferred from previous generations, such as grandparents, parents, family members, or relative women. These “taboos” relate to the selection of foods during pregnancy due to two objectives: the safety of the baby and the woman’s health. Two sub-themes were presented, as follows:

#### Sub-theme 1: taboos aimed at protecting women’s health and ensuring safe childbirth

Nearly all the women discussed and shared their families’ beliefs about prohibiting women from eating certain foods and fruits because they might affect their health and childbirth safety. The women were willing to comply with these taboos without curiosity. This study found some foods and fruits believed to be linked with spontaneous abortion (e.g., papaya salad, papaya fruit, pineapples, coconut juice), prolonging labor (e.g., eggplant), and pregnancy complications (e.g., durian, cha-om). Moreover, several women believed that pregnant women should not eat lunch, because doing so would result in labor dystocia and prolonged labor. For example, one woman said that her grandmother prohibited her from having lunch to prevent difficult labor.

*“My grandmother says it is taboo to have lunch with her*,* because she is afraid*,* it’ll cause an obstruction of labor. So*,* I need to drink some milk and eat some bread instead.” (Woman in First Trimester*,* Group 1)*.

#### Sub-theme 2: taboos aimed at guaranteeing infant safety

According to cultural taboos about harmful foods for infant health, many women in this study admitted that they favored strong, spicy foods pre-pregnancy. Most of them believed that spicy foods affected infant hair loss and irritated their eyes, so pregnant women should not eat them. Moreover, the women in this study were employees who preferred caffeine and soft drinks. These foods might harm infant health. According to taboos, the women believed that they should avoid all kinds of risky foods during pregnancy. For example, one pregnant woman expressed that she had to drink caffeine during pre-pregnancy due to her work schedule. When she got pregnant, she stopped drinking caffeinated beverages to protect the baby’s health.

*“When I was no pregnant*,* I very much liked to drink caffeinated beverages. When I got pregnant*,* I stopped immediately because of my baby.” (Woman in Second Trimester*,* Group 5)*.

Another woman complained that she had to stop eating spicy foods, which were her favorite taste and cultural food style, because her mother told her would affect her infant’s safety.

*“In the beginning*,* I really wanted to eat spicy foods*,* but my mom stopped me because spicy foods will make my baby feel hot and burned*,* or might even cause abortion.”* (Woman in Second Trimester, Group 4).

### Information-seeking Behavior regarding pregnancy diet

#### Theme 3: limited access to appropriate information about nutrition

The immigrant pregnant women in this study had to deal with many roles, including roles as pregnant women, wives, and employees, with restricted conditions such as language, income, healthcare coverage, work policies, and health information. Thus, information-seeking behavior about nutrition was limited by their small communities, and they exchanged some experiences without clarification through healthcare providers. The following two sub-themes were presented:

#### Sub-theme 1: unclear dietary information from healthcare providers

Many women in this study admitted that the nutrition information they should receive during pregnancy for both themselves and their babies’ remained unclear. Some information was provided roughly without any detail, particularly when it conflicted with their cultural beliefs. They suggested a need for instructional materials that could help them re-read and better understand.

For example, one pregnant woman expressed that she had received conflicting information about harmful foods during pregnancy from nurses and her family. She was still living with confusing information.

*“I once heard the nurses say that I could eat anything*,* but it differed from when my parents prohibited me from eating many types of foods such as pineapples*,* watermelons*,* papaya salad*,* cold water*,* etc. I think I believe the nurses*,* because they take care of many clients.” (Woman in Third Trimester*,* Group 7)*.

Moreover, some women said that they did not clearly understand and needed to read the document again at home. The barriers to communication might also have affected their stress.

*“Sometimes I cannot understand what nurses are saying to me; I need some leaflets to read again at home… The difficulty of understanding makes me more stressed.”* (Woman in First Trimester, Group 2).

#### Sub-theme 2: ease of learning from experiences in informal social networks

This study found that pregnant women mostly lived in ethnic enclaves, even if they had a nuclear family. They might have had a barrier in their inability to read and speak in Thai. Almost all the women would discuss diet through informal social networks, such as with their grandmothers, mothers, other relatives, or peers who had previous pregnancy experiences. However, some suggested from their networks that this was improper and created more food limitations. For example, one pregnant woman said that she valued and respected her grandmother, who had previous experience.

*“I must believe her [grandmother]; I cannot read Thai. When my grandma tells me to eat something*,* I will eat without any argument because she [grandmother] had a lot of experience with this [dietary behaviors during pregnancy].” (Woman in First Trimester*,* Group 1)*.

Another woman stated that her friends at work suggested drinking more milk and eating fruits during early pregnancy.

*“When I still had some nausea and vomiting*,* my friends at work who had advised and encouraged me. They suggested that I should drink more milk and eat fruits*,* even though I could not eat anything at the time. It’s good” (Woman in First Trimester*,* Group 3)*.

Moreover, some of the women preferred searching the internet, particularly on YouTube and video clips on social media. However, they would confirm the content with their social networks again.

#### Theme 4: difficult living conditions in a non-native setting

Almost all the women continued to work during pregnancy, which encouraged them to eat healthy foods while performing work responsibilities and earning income. Thus, healthy foods were meaningful, not only to babies but also to the women’s work lives. Regarding working status, the immigrant pregnant women had time restrictions related to accessing information. Social media was the online community for them. Unfortunately, the women faced language barriers to understanding about the health and safety of foods for their babies. The following two sub-themes were presented:

#### Sub-theme 1: work-related influences on dietary behaviors

Almost all of the pregnant women in this study were still working in small or large industries. Many women described their eating patterns and types of foods related to work responsibilities. Moreover, work provided income and more opportunity to get favorite foods and food variety. On the other hand, work could be driven by eating healthy foods to be healthy. For example, one woman stated, *“I have to eat good foods to be strong to work.” (Woman in First Trimester*,* Group 2)*.

In another example, one pregnant woman perceived the importance of work during pregnancy because she was still able to buy her favorite foods and things.

*“I need a lot of money. If I have a lot of money*,* I will buy whatever I want to eat and do whatever I want to do.” (Woman in Second Trimester*,* Group 4)*.

#### Sub-theme 2: lack of comprehensible language to gain food literacy

Several women disclosed that they had some limitations concerning language. They mostly believed their families and peers about food practices during pregnancy. Even when they searched for information on the internet, they often confirmed it with their informal networks for assurance. Thus, searching the internet for nutrition information took more time than asking someone.

For example, one woman who was an employee in a small industry was expecting her child and had less experience finding healthy food for herself. She recognized that she wasted a lot of time searching for knowledge on the internet, so she preferred to ask her Myanmar friends.

*“Because this is my first child*,* I am concerned that I will feed harmful foods to my child. I looked up a lot of things*,* but there was a lot…a lot of data on the internet and social media. I can’t decide and have recognized that I spend a lot of time at work. I think I asked my friends who had previous experiences for sure and it saved time.” (Woman in Second Trimester*,* Group 5)*.

#### Ecological perspectives on nutrition and food practices among pregnant Myanmar immigrant women

**Fig. 1:**
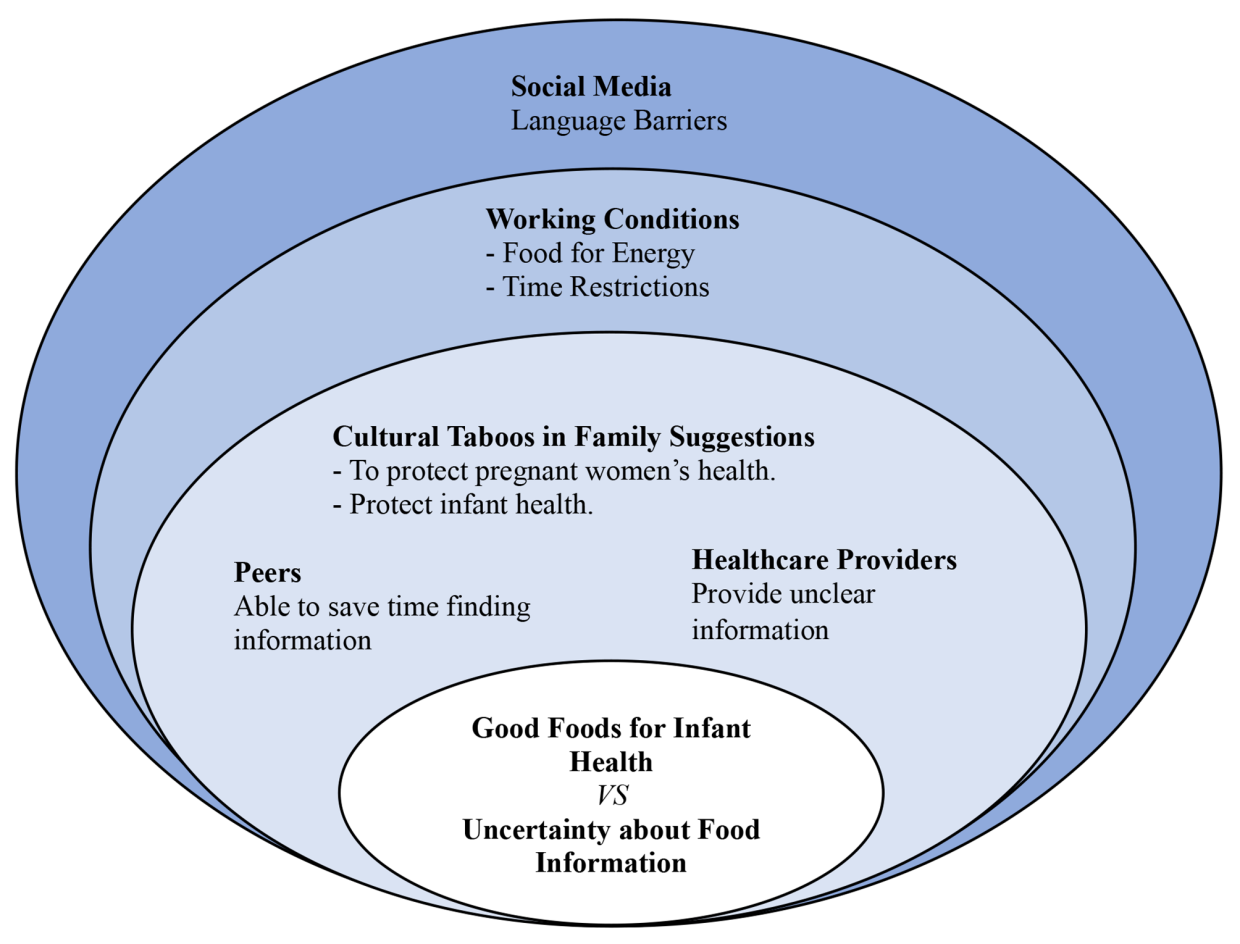
Experiences of Myanmar immigrant women with nutrition and food practices during pregnancy

In addition, the seven focus group discussions (FGDs) involving fifty women highlighted four major themes across four levels of ecological perspectives. The initial theme relied on the intrapersonal level, which disclosed pregnant women’s positive perspectives on changing behavior, while the intrapersonal level was described by family beliefs and a lack of informational support. Moreover, difficult living conditions among working pregnant women and language barriers were shown at institutional and community levels, as presented in Fig. 1.

## Discussion

This study explored the perceptions, beliefs, and information-seeking behaviors concerning nutrition and food practices during pregnancy among Myanmar immigrant women in Thailand. Even though the immigrant women had a positive attitude toward healthy dietary behaviors during pregnancy, the women continued to face several difficulties in achieving healthy food patterns. In particular, the women in this study mentioned facing language barriers, cultural taboos, a lack of information support, and a lack of friendly design to promote nutrition literacy. To improve the quality of nutrition and food practices during pregnancy among vulnerable groups, the ecological system should involve multiple levels, including family, peer group, workplace, hospital, and community levels, as well as social innovation.

We found a positive attitude toward dietary practices among this group since pregnancy due to concern about their child’s health. Even though the women had restricted access to food resources, they mentioned eating healthy foods by increasing protein and carbohydrates in addition to avoiding risky foods. Moreover, this study showed that nearly half of the women had had a high BMI (≥ 23 kg/m2) before pregnancy. However, the women were not aware of food variety, safety, or quantity. According to a study of nutrition in pregnant women on the Thailand–Myanmar border, high BMI increased markedly from 16.9 to 33.2% for refugees and 12.3 to 28.4% for migrants over 13 years (2004–2016) [31]. Similarly, migrant women living in urban areas tended to eat high-calorie-dense and nutrition-poor fast foods during pregnancy [14], while one study in a high-income country reported that immigrant women in Canada mentioned the quality of foods and the importance of “fresh foods,” adding healthy foods such as vegetables, proteins, or milk [46]. Despite constraints in accessing expensive ingredients, obtaining appropriate information for selecting nutritious foods, and obtaining adequate quantities for pregnant women from safe and affordable food sources, the above should be an available option for migrant pregnant women.

Importantly, the women were obstructed by unclear nutrition information and education in the antenatal care unit as well as social media platforms. The language barrier was a vital problem for increasing effective health communication. Although the Thai Government started implementing “migrant-friendly health services” through interpretation and cultural mediation services in 2003, operational challenges in providing services included insufficient budgets for employment and training, diverse training curricula, and a lack of legal provisions to sustain the programs [47]. Moreover, some women preferred to use social media. According to a previous study, French pregnant women had nutrition-related information that was perceived as contradictory, which led to confusion that could limit the adoption of healthier eating behaviors [48]. To address the health needs of migrant pregnant women, clear policy regulation is required and lead agencies should be mandated to collaborate with stakeholders (government, NGOs, employers, migrant pregnancy, and healthcare providers) in planning overall structure and resource allocation.

The Myanmar cultural beliefs about foods during pregnancy include several taboos associated with food choices and patterns, leading to some stressful situations dealing with the amplification of food cravings. Previous studies have shown specific cultural beliefs related to nutritional behavior and the overall health status of women and children. In Chinese culture, people think black and red foods are good for our body, especially the blood. In South Asian culture, women believe in the importance of drinking liquids for ease of delivery [46]. However, some women are prohibited from eating certain foods because of the impact of stress on eating behaviors [49, 50]. The ethno-cultural socialization and experiences of women highly influence their antenatal behaviors, especially in terms of food consumption patterns [46]. Moreover, a study of indigenous pregnant women in Ecuador revealed common characteristics that were important challenges for nutritional behavior during pregnancy and the health system: illiteracy, low income, and women’s age during pregnancy [51]. Inadequate intake of nutrients and micronutrients during pregnancy may result from avoiding foods that are high in nutrition, such as meat, dairy, and some fruits and vegetables. Pregnant women who have food taboos may be more vulnerable to poor nutrition and pregnancy-related problems [52, 53]. As a result, pregnant women frequently experience various levels of nutritional stress, and those who adhere to traditional food taboos are more likely to experience a variety of adverse pregnancy outcomes, such as negative effects on infant health in the future [54]. Healthcare professionals must, therefore, be aware of the cultural taboos that could raise the possibility of poor pregnancy outcomes.

In addition, this study found a significant influence of working status on food consumption patterns and choices in terms of the benefits of working energy, limited time for food preparation, and income allowing the women access to healthy food choices. However, this result differed from a previous study that found adherence to food patterns in pregnant Brazilian women to be associated with working inside the home or being unemployed [55]. Therefore, employers are an important stakeholder in facilitating a healthy and safe food environment for migrant pregnant women who continue to work during pregnancy.

Pregnancy is a crucial time in which maternal outcomes and fetal development depend on optimal nutrition [1]. Immigrant pregnant women are a vulnerable population that should be provided with equality to ensure quality of life for both women and their children. To optimize Myanmar immigrant pregnant women through improved nutrition and food practices during pregnancy, the ecological system should be comprehensively implemented in supporting and enforcing healthy eating and behaviors. However, this study had some limitations in the data collection process, which included interviews with Thai researchers, which might have obstructed some flow of group discussion, even though interpreters were present. Dietary behaviors should be designed with triangulation in terms of observation and embedded in the context of this group. Moreover, this study did not mention the socioeconomic and working details potentially affecting decisions on dietary behaviors. The implication is that practice and policy require collaboration with all stakeholders in policy involvement in planning “migrant-friendly health services” to eliminate language barriers and combine nutrition literacy among immigrant pregnant women. Moreover, healthcare providers should create effective interventions to communicate appropriate nutrition information with immigrants and their families, along with cultural sensitivity. Nutrition literacy innovation may be needed to translate health information and facilitate decision-making for migrant pregnant women. Furthermore, for many pregnant Myanmar immigrant women, implementing culturally appropriate nutritional guidelines may be an essential part of antenatal care. Concerned stakeholders should create comprehensive and contextual educational programs that address common causes (myths and misconceptions) of pregnancy-related food taboos and motivate women to consume essential nutrients for good health. At the policy level, we propose that prenatal health promotion initiatives prioritize equity for pregnant immigrant women with a focus on nutritious food to optimize positive pregnancy outcomes. In order to provide proper support for pregnant women from different cultures and their families, healthcare professionals should receive training in cultural competency and sensitivity.

## Conclusion

This study found multilevel influences on nutrition and food practices during pregnancy among Myanmar immigrant women, who disclosed their experiences concerning dietary practices, which had important changes compared to the pre-pregnancy period. Meanwhile, the immigrant’s life contexts and cultural beliefs significantly affected their adaptations and decisions about food practices. They were willing to change their harmful nutrition behaviors to suitable ones for the safety of their infants. However, the women continued to lack nutrition knowledge, literacy, and accessibility information, as well as support from healthcare providers. Additionally, they encountered difficulties with restricted food culture, work characteristics, and communication that inhibited food insecurity during pregnancy. Healthcare providers should develop nutrition and health innovations embracing cultural sensitivity in order to improve pregnancy outcomes and quality of life among pregnant migrant women.

## Data Availability

No datasets were generated or analysed during the current study.
